# Physical activity for antenatal and postnatal depression in women attempting to quit smoking: randomised controlled trial

**DOI:** 10.1186/s12884-018-1784-3

**Published:** 2018-05-10

**Authors:** Amanda Daley, Muhammad Riaz, Sarah Lewis, Paul Aveyard, Tim Coleman, Isaac Manyonda, Robert West, Beth Lewis, Bess Marcus, Adrian Taylor, Judith Ibison, Andrew Kent, Michael Ussher

**Affiliations:** 1 Sport, Exercise and Health Sciences, Loughborough University, Epinal Way, Loughborough, Leicestershire LE11 3TU UK; 2 Population Health Research Institute, University of Leicester, University Road, Leicester, LE1 7RH UK; 3 Division of Epidemiology and Public Health and UK Centre for Tobacco and Alcohol Studies, University of Nottingham, Nottingham, NG5 1PB UK; 4 Nuffield Department of Primary Care Health Sciences, Radcliffe Observatory Quarter, University of Oxford, Woodstock Road, Oxford, OX2 6GG UK; 5 Division of Primary Care and UK Centre for Tobacco and Alcohol Studies, University of Nottingham, Nottingham, NG5 1PB UK; 6 Department of Obstetrics and Gynecology, St George’s University of London and St George’s NHS Trust, Blackshaw Road, London, SW17 0QT UK; 7 Health Behaviour Research Centre, Department of Epidemiology and Public Health, UCL, Gower Street, London, WC1E 6BT UK; 8 School of Kinesiology, University of Minnesota, Minneapolis, MN 55455 USA; 9 Department of Family and Preventive Medicine, University of California, San Diego, CA 92093-0628 USA; 10 Plymouth University Peninsula Schools of Medicine and Dentistry, Plymouth Science Park, Plymouth, Devon PL6 8BX UK; 11 Institute of Medical and Biomedical Education, St George’s University of London, Cranmer Terrace, London, SW17 ORE UK; 12 Population Health Research Institute, St George’s University of London, Cranmer Terrace, London, SW17 ORE UK; 13 Institute for Social Marketing, University of Stirling, Stirling, Stirling, FK9 4LA UK

**Keywords:** Pregnancy, Physical activity, Antenatal, Postnatal, Depression

## Abstract

**Background:**

Antenatal depression is associated with harmful consequences for both the mother and child. One intervention that might be effective is participation in regular physical activity although data on this question in pregnant smokers is currently lacking**.**

**Methods:**

Women were randomised to six-weekly sessions of smoking cessation behavioural-support, or to the same support plus 14 sessions combining treadmill exercise and physical activity consultations.

**Results:**

Among 784 participants (mean gestation 16-weeks), EPDS was significantly higher in the physical activity group versus usual care at end-of-pregnancy (mean group difference (95% confidence intervals (CIs)): 0.95 (0.08 to 1.83). There was no significant difference at six-months postpartum.

**Conclusion:**

A pragmatic intervention to increase physical activity in pregnant smokers did not prevent depression at end-of-pregnancy or at six-months postpartum. More effective physical activity interventions are needed in this population.

**Trial registration:**

Current Controlled Trials ISRCTN48600346. The trial was prospectively registered on 21/07/2008.

## Background

Ten to 20 % of pregnant women become depressed during or after pregnancy [1, 2]. Antenatal depression is associated with harmful consequences for the mother and child, including premature labour, low birth weight, and a compromised mother-child relationship [3–5]. Postnatal depression can adversely affect children’s cognitive and emotional development and social behaviour [6]. Antidepressants are effective for treating depression outside of pregnancy [7], but are seldom prescribed during pregnancy due to concerns about potential adverse effects on the fetus or infant [8]. Psychological therapies can be effective [9], but there can be long waiting lists to access treatment and they can be costly. It is important to find alternative effective interventions. One intervention that might be effective is participation in regular physical activity. Thirty minutes of moderate intensity physical activity per day is recommended for mental health benefits during pregnancy [10–13]. However, this guidance is based on studies with non-pregnant individuals and may have limited applicability to pregnant and postnatal populations.

The evidence for using physical activity to prevent or treat depression in pregnancy is mixed: meta-analyses provide evidence for physical activity interventions reducing depression scores both during pregnancy (six trials, *n* = 364) and postnatal (five trials *n* = 221) [14, 15], however, there was significant heterogeneity between trials. Also, the reviewed studies were judged to be of poor to moderate quality. A more recent meta-analysis of antenatal interventions to reduce maternal distress, including depression, did not consider any physical activity trials as being rigorous enough to be included [16]. Further trials that postdate these systematic reviews showed no benefit of physical activity for preventing antenatal or postnatal depression, [17, 18]. Subsequent trials however have showed a benefit for an exercise intervention for reducing depression during pregnancy and postnatally, although one study did not report exercise adherence [19] and the other failed to show a significant difference in physical activity between the intervention and usual care groups at follow up [20]. There is a need, therefore, for more robust evidence. The recently completed London Exercise and Pregnant Smokers (LEAP) trial investigated whether behavioural support to enhance a woman’s physical activity at home, combined with twice-weekly supervised exercise sessions, would increase rates of continuous and biochemically validated smoking abstinence at end of pregnancy [21]. The main rationale for physical activity aiding smoking cessation is the evidence for exercise reducing cigarette cravings and withdrawal symptoms. [22, 23] The LEAP intervention was ineffective at aiding cessation [24].

As a secondary outcome, the present study assessed the effect of the LEAP intervention on antenatal and postnatal depression, in the largest randomised controlled trial (RCT) to date. The outcomes were change in depression from baseline to end-of-pregnancy and six months after birth. In the UK, approximately 12% of pregnant women smoke throughout pregnancy [25] and these women are at heightened risk of depression during pregnancy [26]. We hypothesised that exercise counselling and provision of regular supervised exercise would reduce depression compared with usual care.

## Methods

### Participants

Pregnant women were recruited via 13 hospital antenatal clinics across the UK. All the hospitals were in localities that included areas of deprivation and social housing. The inclusion criteria for the trial were a desire to stop smoking, wanting help to stop smoking, agreeing to set a date for quitting smoking within one week of the baseline assessment, aged 16-50 years, being between 10 and 24 weeks of gestation, cigarette consumption of five or more daily before pregnancy, currently smoking one or more cigarettes per day, and being able to walk continuously for at least 15 min. Women were not eligible if they had health conditions that had potential to be exacerbated by exercise or advised not to exercise by a medical doctor, unable to provide informed consent or complete the study questionnaires in English, dependence on drugs or alcohol, and currently using or wanting to use nicotine replacement therapy. Written informed consent was obtained from all participants.

### Design and interventions

This study reports analysis of depression outcomes from a RCT evaluating the effect of a physical activity intervention on smoking cessation during pregnancy [21]. At the first session, participants were randomly assigned (based on a computer-generated code) to either behavioural cessation support alone (usual care) or to this support plus a physical activity intervention. In the primary analysis, the rates of continuous biochemically validated smoking cessation at end of pregnancy were similar in the physical activity group (7.7%) and usual care group (6.4%) [24].

#### Intervention

Participants were offered the opportunity to take part in six weekly sessions of 20 min of individual behavioural support for smoking cessation, starting one week before the smoking quit date and ending four weeks later. This intervention seeked to support smoking cessation by reinforcing commitment to abstinence and solving participants’ problems about maintaining smoking abstinence. The intervention also aimed to improve the mental health of women.

The physical activity intervention combined supervised exercise sessions with physical activity consultations. All sessions were individual and took place in a private room at the hospital or in a community health centre or children’s centre. Fourteen sessions of supervised exercise were offered over eight weeks; twice a week for six weeks, followed by weekly sessions for two weeks. At each session participants were asked to walk at a moderate intensity on a treadmill for up to 30 min. At the first two treadmill sessions, and then on alternate occasions (total of nine consultations), they received the physical activity consultations, which aimed to identify opportunities to incorporate physical activity into women’s daily lives and to help them use behavioural strategies to improve adherence to these plans. These 20 min consultations incorporated 19 behaviour change techniques, as previously described in the protocol [21]. Participants were advised to be active for at least 10 bouts, progressing towards 30 min of activity on at least five days a week, with an emphasis on brisk walking. As a motivational tool (not a research measure), participants were given a pedometer (Digi-Walker SW-200; Yamax, Nottingham, UK) and were encouraged to record the number of steps they had achieved each day, with the researcher calculating a 10% increment every two weeks; the overall goal being to work towards accumulating 10,000 steps per day [27]. Participants received £7 for their travel expenses for each session that they attended and were given a DVD on antenatal exercise. On the other occasion the women received behavioural support for smoking sessions (up to six sessions) as for the usual care group.

### Measures

One week prior to the quit date, baseline data was collected for demographic variables, smoking characteristics and physical activity behaviours, including the Fagerström Test for Cigarette Dependence score (FTCD, [28, 29]) and self-reported levels of moderate and vigorous intensity physical activity (MVPA) in the previous week using a seven-day physical activity interview [30]. Self-reports of physical activity included all types of activity, irrespective of context (e.g., leisure, occupational). Depression was measured with the Edinburgh Postnatal Depression Scale (EPDS [31], via a face-to-face consultation at baseline and at end of pregnancy and via telephone at six months after the birth. The EPDS is a self-report 10-item scale (each is scored 0-3, range = 0 to 30) designed to assess antenatal and postnatal depression in community samples [32], is widely used and has been validated in many countries [33]. It can be used as a continuous outcome or to classify women as probable cases of antenatal/postnatal depression at a cut-off of 13 or above [34]. Further self-reports of physical activity levels were collected at weeks one, four, and six after the quit date, at end of pregnancy and six months after the birth. In an 11.5% random subsample of participants (the target was 10%) physical activity was objectively measured using an accelerometer (Model GT1M or GT3X; Actigraph, Pensacola, FL, USA). This was worn over the right hip, in the fourth week after the quit date, for seven consecutive days, recording ‘dry land’ activity during waking hours at one minute epochs.

### Analysis & Sample Size

Details of the sample size calculation can be found in previous publications. [21, 24] First, we checked whether those providing EPDS data at the two follow-ups (end-of-pregnancy, six months postnatal) had similar baseline characteristics and physical activity at follow-up as the whole sample. Then we examined whether the baseline characteristics of the physical activity versus usual care group were similar in the sub-samples with EPDS data at the two follow-ups.

For the primary analysis EPDS data was treated as a continuous variable. We used a mixed-effect linear model with EPDS scores at end-of-pregnancy and six months postnatally as dependent variables. To estimate the difference between physical activity and usual care groups at each follow-up adjusting for baseline EPDS and recruitment centre, we fitted a linear mixed effect model including as independent variables the interaction of treatment groups and follow-up times, follow-up times, recruitment centre and the interaction of baseline EPDS score with follow-up times. The model accounts for within-person correlation over time and assumes that data is missing completely at random for the participants with missing EPDS data at follow-ups. In the next step, we further adjusted for the following potential predictors of postnatal depression: marital status, age at leaving full time education (as a proxy for socioeconomic status), body mass index and young age (i.e., age <  20 years). At end-of-pregnancy about half of the women (201/383, 52.5%) provided EPDS data before the birth and half after the birth (182/383, 47.5%); therefore we examined the difference between the mean EPDS scores before and after the birth using t-tests.

As a secondary analysis EPDS was treated as a binary variable. We assessed whether the proportion reaching the EPDS cut-off of ≥ 13 for depression changed at the follow-ups, for physical activity versus usual care, relative to baseline. We used a mixed effect logistic regression model, with the same independent variables as before, to estimate the adjusted odds ratios (OR) of depression for physical activity group versus usual at end-of-pregnancy and 6 month follow-up. This analysis was further adjusted for the same predictors used in the analyses of EPDS as a continuous variable. One recommendation is to use a EPDS cut-off of ≥ 15 antenatally and ≥ 13 postnatally [35], therefore we conducted a further analysis using ≥ 15 for baseline and end-of-pregnancy follow-up before the birth, and ≥ 13 for end-of-pregnancy after the birth. Additionally, in order to explore whether adverse events might have influenced the EPDS outcomes we report these events for the two study groups.

We used multiple imputation analysis to explore the impact of missing data Missing values in the EPDS score at follow-up were replaced by imputed values using chained equations [36] with the predictive mean matching method on the basis of the baseline explanatory variables of EPDS scores at baseline, randomisation groups, body mass index, carbon monoxide level, smoking abstinence at end-of-pregnancy, self-reported minutes of physical activity at baseline, age at leaving full-time education, gestational age (weeks) at baseline, number of cigarettes smoked before pregnancy, number of cigarettes smoked at baseline, FTCD score, marital status, ethnicity, type of physical activity at baseline, parity, partner smoking status, self-efficacy, confidence for physical activity, recruitment centre, perceived positive effects of being physically active. We created twenty imputed datasets and used the same model as above to estimate the treatment effects on EPDS score in these datasets. We combined the imputation-specific estimates using Rubin’s rules [37]. All statistical analyses were performed using Stata (version 12).

To explore whether the effect of treatment on depression scores differed according to how physically active the women were at baseline, we tested for an interaction between baseline physical activity (< 150 min/week MVPA versus ≥ 150 min/week MVPA) and the treatment effect for the primary outcome of EPDS score at both follow-ups.

We also explored whether those who adhered better to the physical activity regime had a greater response to treatment by looking at modification of the treatment effect according to whether the individual reported ≥ 150 min/week MVPA at four weeks or six weeks after the quit day in those reporting < 150 min/week MVPA at baseline.

## Results

**Fig. 1:**
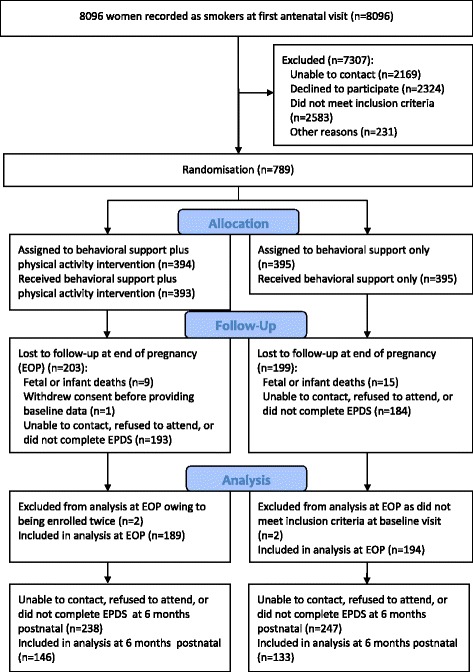
Numbers of participants who were enrolled in the study and included in the analyses for Edinburgh Postnatal Depression Scale score (EPDS). The participants lost to follow-up included some who had fetal or infant loss and were not assessed for EPDS score

Between April 2009 and November 2012 a total of 789 participants were recruited and randomised. This was 10% of the women recorded as smokers at their first antenatal booking visit (see Fig. 1). Four women were excluded after randomisation; two women in the intervention group were randomised twice in sequential pregnancies and their second enrolment was removed, and two participants in the usual care group were ineligible at their baseline visit and had been randomised erroneously. When using an intention to treat approach it is acceptable to exclude patients’ data, without risking bias, when ineligible patients are mistakenly randomised into a trial [38]. One participant was randomised but withdrew consent without giving any reason, before providing any data. A total of 784 women were randomised and included in this study.

EPDS data was provided by all 784 participants at baseline; by 383 (49%) at the end of pregnancy, and by 279 (36%) at six-months postnatally (see Fig. 1). The baseline characteristics of those with EPDS data available at end-of-pregnancy and six months were similar to those of recruits as a whole (see Table 1). The baseline characteristics of the trial groups were also very similar and these have been reported elsewhere [24]. In addition, the baseline characteristics and physical activity levels at follow-up were similar for women who did and did not report on their depression.

**Table 1:** Characteristics for participants in the total trial sample and in the sub-samples providing EPDS data at end-of-pregnancy and 6 months postnatally

	Total sample *n* = 784	End-of-pregnancy *n* = 383	6 months *n* = 279
	Mean (SD)	Mean (SD)	Mean (SD)
Age	27.5 (6.3)	27.7 (6.2)	28.1 (6.2)
Age left full-time education	17.7 (2.9)	17.9 (2.8)	18.0 (2.9)
Weight at booking – kg	68.3 (15.2)	68.8(15.4)	68.9 (15.9)
Body Mass Index kg/m^2^	26.1 (5.3)	26.0 (5.2)	26.1 (5.5)
Gestational age – wk	15.6 (3.3)	15.3 (3.2)	15.4 (3.1)
	Median (IQR)	Median (IQR)	Median (IQR)
Fagerström Test of Cigarette Dependence score	4.0 (2.0-5.0)	4.0 (2.0-5.0)	4.0 (2.0-5.0)
EPDS score at baseline	7.0 (3.5-11.0)	8.0 (4.0-12.0)	7.0 (4.0-11.0)
Self-report of weekly MVPA	210.0	210.0	210.0
– mins (at baseline)	(130.0-355.0)	(125.0-330)	(120.0-340.0)
Self-report of weekly MVPA	150.0	150.0	140.0
– mins (at EOP)^a^	(75.0-240.0)	(75.0-240.0)	(70.0-240.0)
Self-report of weekly MVPA	162.5	165.0	155.0
– mins (at 6 months follow up)	(60.0-300.0)	(80.0-310.0)	(60.0-300.0)
Treatment sessions attended^a^	3 (2-6)	6 (3-7)	5(3-7)
	n (%)	n (%)	n (%)
Allocation to physical Activity group	392 (50)	189 (49)	140 (52)
IMD^b^ quartiles 3 or 4	555 (74)	259 (71)	191 (72)
Married or living with partner	451 (58)	224 (59)	181 (65)
Caucasian	607 (77)	301 (79)	226 (81)
Professional/managerial occupation	99 (13)	48 (13)	39 (14)
EPDS score at baseline ≥ 13	143 (18)	51 (13)	29 (10)
Thought of harming oneself (EPDS)	20 (3)	11 (3)	7 (3)
Self-report of ≥ 150 mins week MVPA (baseline)	548 (70)	266 (70)	185 (66)
Parity (range = 0 to 5)			
0-1	606 (78)	301 (79)	220 (79)
2-3	155 (20)	75 (20)	54 (19)
≥ 4	21 (3)	7 (2)	5 (2)
Takes alcohol ≥ twice a week	11 (1)	6 (2)	5 (2)
Consumes ≥ 3 alcoholic drinks on a drinking day	17 (10)	7 (8)	6 (10)

*EPDS* Edinburgh Post-natal Depression Scale, *EOP* End of pregnancy, *IMD* Index of Multiple Deprivation, *MVPA* Moderate and vigorous physical activity. ^a^Non-baseline measure ^b^*N* = 752 as there were 12 missing postcodes and 20 invalid post codes

Participants attended a median (IQR) of four (2-8) treatment sessions in the intervention group and three (2-6) in the usual care group. As reported previously [24], for the physical activity group, compared with the usual care group, the self-reported minutes of MVPA were significantly higher by 33% (95% CI: 14, 56%), 28% (7, 52%), and 36% (12, 65%) at one week, four weeks and six weeks, respectively; while among the 11% of participants wearing an accelerometer MVPA levels were very similar for the two study groups.

### Primary analysis

EPDS score was significantly higher in the physical activity group versus the usual care group at end-of-pregnancy with both the basic adjustments and in the fully adjusted model (see Table 2). At this time, there was mean increase in EPDS score of 0.4 in the physical activity group and a mean reduction of 0.5 in the usual care group (mean difference between groups (95% CIs) in fully adjusted model: 0.95 (0.08 to 1.83)). At end-of-pregnancy, the EPDS scores did not differ significantly between women who provided data before birth and those who provided after birth. There was no significant difference in EPDS score between the groups at six-months postnatal (fully adjusted mean difference (95% CIs): 0.37(− 0.59 to 1.33)). There was no significant interaction between baseline self-reports of MVPA (< 150 vs ≥ 150 min/week physical activity) and the treatment effect at either time point (at end-of-pregnancy: *p* = 0.346; at 6 months follow up: *p* = 0.607); similar results were found for the fully adjusted model. At end-of-pregnancy, the effect of physical activity was different in those who did and those who did not increase their exercise between baseline and four weeks post quit; for those who did increase, the estimated difference between (95% CI) physical activity versus control was 3.48 (1.46 to 5.51), *p* = 0.001, and for those who did not increase it was 1.14 (− 0.60 to 2.89), *p* = 0.199, but these differences were not significant at 6 months follow-up.

**Table 2:** Comparison^a^ of depression scores in two trial groups at end-of-pregnancy and six months after birth (*N* = 784^d^)

Visit time	EPDS score N, Mean (SD)		β (difference between PA and usual care, basic adjustment^b^) (95% CI)	β (difference between PA and usual care group, fully adjusted^c^) (95% CI)
	Usual care group	Physical activity group		
baseline	393, 7.7 (5.0)	391, 7.6 (5.3)		
End of pregnancy	194, 7.2 (5.0)	189, 8.0 (4.9)	1.06 (0.19, 1.94) *p* = 0.017	0.95 (0.08, 1.83) *p* = 0.033
6 months follow-up	133, 6.6 (4.7)	146, 6.8 (4.8)	0.52 (−0.45, 1.50), *p* = 0.293	0.37 (−0.59, 1.33) *p* = 0.450

*EPDS* Edinburgh Postnatal Depression Scale, *PA* Physical activity

^a^Results from mixed effect linear model

^b^Adjusted for visit time, baseline EPDS, recruitment centre, the interaction of baseline EPDS and visit time

^c^Adjusted for visit time, baseline EPDS, recruitment centre, the interaction of baseline EPDS and visit time, age at leaving full time education, young age (≤ 20 yrs), BMI, marital status

^d^For the final mixed effect model, *N* = 436

### EPDS data as a binary outcome

When treating the EPDS score as a binary variable (i.e., using a cut-off score of ≥13) there was no significant difference between the groups at either follow-up point, although the ORs showed a slightly higher risk of probable depression in the physical activity versus usual care group (fully adjusted OR (95% CIs): 1.11 (0.47 to 2.65; 1.07 (0.36 to 3.17), for end-of-pregnancy and six months, respectively) (see Table 3). Similar results were produced when using an EPDS cut-off of ≥15 at baseline and end-of-pregnancy (before birth), and ≥ 13 at end-of-pregnancy (after birth).

**Table 3:** Comparison^a^ of EPDS score indicating presence or absence of depression in trial groups at end-of-pregnancy and six months after birth (*N* = 784)

Visit time	Depression in each randomisation group		Odds Ratio for PA versus usual care, basic adjustment^b^ (95% CI)	Odds Ratio for PA versus usual care, fully adjusted^c^ (95% CI) (95% CI)
	Usual care n/N (%)	Physical Activity n/N (%)		
Baseline	75/393 (19)	68/391 (17)	–	–
End of pregnancy	25/194 (12)	26/189 (14)	1.24 (0.52, 2.96) *p* = 0.628	1.11 (0.47, 2.65) *p* = 0.808
6 months follow-up	14/133 (11)	15/146 (10)	1.23 (0.41, 3.70) *p* = 0.710	1.07 (0.36, 3.17) *p* = 0.904

*EPDS* Edinburgh Postnatal Depression Scale, *PA* Physical activity

^a^Results from mixed effect logistic model for depression (yes = 1, No = 0)

^b^Adjusted for visit time, baseline EPDS scores, the interaction of visit time and baseline EPDS, and recruitment centre

^c^Adjusted for visit time, baseline EPDS scores, the interaction of visit time and baseline EPDS scores, recruitment centre, age at leaving full time education, young age (≤ 20 yrs), BMI, marital status

For the final mixed effect model, *N* = 436

When we used multiple imputation as an alternative way of dealing with missing data the results were very similar; in particular, the fully adjusted mean difference (95% CIs) in EPDS score between treatment and control groups at end-of-pregnancy was 0.98 (0.10 to 1.85), *p* = 0.029 and at 6 months it was 0.33 (− 0.71 to 1.38), *p* = 0.526.

The rate of at least one adverse event was very similar for the two trial groups (217 (55.5%), 219 (55.7%), in the physical activity and usual care groups, respectively).

## Discussion

### Main findings

Among pregnant women there was significantly increased depression scores at the end of pregnancy but there was no difference six months after birth. When depression was considered as a binary outcome (those with and without probable depression) there was no evidence of a difference between treatment arms at either follow up. These results emerged despite the physical activity group reporting significantly more minutes of moderate and vigorous physical activity throughout pregnancy.

Our findings are consistent with two trials observing that physical activity did not reduce depression scores/prevalence before or after pregnancy [17, 18], but differ from meta-analyses and recent trials showing benefits for physical activity on both antenatal and postnatal depression [14, 15, 19, 20]. However, there were methodological limitations in these studies and this raises the possibility that the effect of physical activity on depression could have been over-estimated by these trials. This study joins other well conducted trials that have recruited non-pregnant populations which have reported that exercise interventions do not improve depression outcomes [39].

### Interpretation in light of other findings

Several explanations are possible for our findings. Women recruited were more active than average, perhaps because active women were attracted to a trial promoting physical activity. This could suggest a ceiling effect where provision of behavioural support to increase activity was ineffective in already active women. We examined whether the effect on depression was greater in women who were inactive at baseline and found no evidence among this subgroup, but there was evidence that depression worsened among women who became active in the intervention group. Taken together, these results suggest that data remain at odds with that from other trials which tend to point towards a benefit from exercise, not an adverse effect, as here.

An alternative explanation is that the difference in the increase in physical activity between the study groups may be less than indicated by the self-reported results and this is supported by the accelerometer results which suggested that physical activity levels were similar in the two groups. Thus, it is possible that the physical activity group did not truly increase their physical activity, as intended. A further explanation relates to the population of interest and the requirements of the intervention. Those in the intervention group were asked to change two health behaviours simultaneously (i.e., smoking and physical activity) while also coping with being pregnant, in addition to dealing with the demands of being asked to attend multiple treatment sessions. These demands might have demoralised some individuals, and they may have found this difficult to achieve, resulting in marginally higher depression scores at the end of pregnancy. It is also possible that treadmill walking/gym based exercise may not be the ideal mode of physical activity and women may well have benefitted more from activity undertaken outside.

### Strengths and limitations

The mental health of pregnant smokers is an important clinical question. There has been no other adequately powered trial of pregnant women and exercise, so this study adds new knowledge. This study included a large sample of women (*N* = 784) and the prevalence of probable cases of depression at baseline and follow-up in the usual care group was marginally higher than typical prevalence levels reported in the literature [40], which would be expected among smokers, and this maximises our ability to detect differences due to treatment. Participants were recruited directly from several hospitals in the UK at their antenatal appointment (typically 10-12 weeks of pregnancy) and a large proportion were of non-white ethnicity (23%) and/or living in the two highest IMD deprivation quartiles (74%). This is important as these populations of women can be difficult to recruit and they are the groups at greatest risk of depression, again maximising the ability to detect treatment effects. Attendance at treatment sessions was modest but this was a pragmatic trial of a behavioural intervention that was conducted in a ‘real life’ NHS setting and women in the intervention group still reported significantly higher physical activity scores than those receiving usual care. Other high quality trials^39^ that have had modest to good adherence have also failed to demonstrate an effect for exercise on depression, so our findings may be unrelated to session adherence. We did not include an objective assessment of physical activity behaviour in all women and as previously observed [41], accelerometer validation suggests that participants overestimated their self-reported physical activity.

Less than half the women enrolled provided data on depression at both follow-up points. The relatively low follow-up rates may be because women who have relapsed to smoking are often reluctant to attend further appointments. Loss to follow-up could have introduced bias but women who gave data appeared to have no systematic differences from the whole population. Also, the findings were very similar when using the imputed data. Nonetheless it is unlikely that loss to follow up led to the non-significant effects because we would have to assume that women who failed to be followed up showed a marked improvement in mood while those who remained in the study showed no change or worsening and usually the opposite is the case.

This study reports an outcome from a trial that was powered to detect the effects on the primary outcome of the trial (i.e., smoking abstinence rates) and power calculations were not conducted for the secondary endpoint of EPDS scores. Depression scores did not differ significantly between the groups at six months postnatal follow-up and it is possible that the trial was insufficiently powered to detect this. However, neither the small effect size, nor those indicated as potential effect sizes on the basis of the 95% confidence interval, would be of clinical relevance. Thus, it seems unlikely that lack of power explains the results at six months follow up.

## Conclusion

At the end of pregnancy the physical activity group did not report significantly lower depression scores than usual care; in fact the intervention group reported significantly higher depression scores, although the magnitude of this difference was small and unlikely to be clinically relevant [34, 42]. And when treating depression as a binary outcome there were no differences between the two study group. Depression scores did not differ significantly between the groups six months postnatally either. While clinical guidelines recommend that pregnant women exercise for mental health benefits and it is recommended that physical activity be used for treating depression among smokers, the pragmatic facilitated physical activity intervention used in the LEAP trial cannot be recommended as treatment or prevention for antenatal or postnatal depression in pregnant smokers. The lack of a beneficial effect of the intervention on depression scores could be partly explained by the possibility that intervention group did not increase their activity levels sufficiently relative to the usual care group, as reflected in the two groups having similar activity levels among the sub-sample with objectively measured physical activity. This suggests that more effective physical activity interventions may be needed in this population, rather than that physical activity cannot be recommended for moderating depression during pregnancy and postpartum.
